# Mycotoxin Biodegradation Ability of the Cupriavidus Genus

**DOI:** 10.1007/s00284-020-02063-7

**Published:** 2020-06-05

**Authors:** Mohammed AL-Nussairawi, Anita Risa, Edina Garai, Emese Varga, István Szabó, Zsolt Csenki-Bakos, Balázs Kriszt, Mátyás Cserháti

**Affiliations:** 1 Department of Environmental Safety and Ecotoxicology, Faculty of Agricultural and Environmental Sciences, Szent István University, 1 Páter Károly Street, Gödöllő, 2100 Hungary; 2 Department of Aquaculture, Faculty of Agricultural and Environmental Sciences, Szent István University, 1 Páter Károly Street, Gödöllő, 2100 Hungary; 3 Department of Applied Chemistry, Faculty of Food Sciences, Szent István University, Villanyi Road, Budapest, 1118 Hungary

## Abstract

The biodegradation and biodetoxification ability of five prominent mycotoxins, namely aflatoxin B1 (AFB1), ochratoxin-A (OTA), zearalenone (ZON), T-2 toxin (T-2) and deoxynivalenol (DON) of *Cupriavidus* genus were investigated. Biological methods are the most appropriate approach to detoxify mycotoxins. The *Cupriavidus* genus has resistance to heavy metals and can be found in several niches such as root nodules and aquatic environments. The genus has 17 type strains, 16 of which have been investigated in the present study. According to the results, seven type strains can degrade OTA, four strains can degrade AFB1, four strains can degrade ZON and three strains can degrade T-2. None of the strains can degrade DON. The biodetoxification was measured using different biotests. SOS-chromotest was used for detecting the genotoxicity of AFB1, the BLYES test was used to evaluate the oestrogenicity of ZON, and the zebrafish embryo microinjection test was conducted to observe the teratogenicity of OTA, T-2 and their by-products. Two type strains, namely *C. laharis* CCUG 53908^T^ and *C. oxalaticus* JCM 11285^T^ reduced the genotoxicity of AFB1, whilst *C. basilensis* DSM 11853^T^ decreased the oestrogenic of ZON. There were strains which were able to biodegrade more than two mycotoxins. Two strains degraded two mycotoxins, namely *C. metalliduriens* CCUG 13724^T^ (AFB1, T-2) and *C. oxalaticus* (AFB1, ZON) whilst two strains *C. pinatubonensis* DSM 19553^T^ and *C. basilensis* degraded three toxins (ZON, OTA, T-2) and *C. numazuensis* DSM 15562^T^ degraded four mycotoxins (AFB1, ZON, OTA, T-2), which is unique a phenomenon amongst bacteria.

**Electronic supplementary material:**

The online version of this article (10.1007/s00284-020-02063-7) contains supplementary material, which is available to authorized users.

## Introduction

The genus *Cupriavidus* was identified in 2004 [1]. Members of this genus are gram negative, chemoorganotrophic and facultative chemolithotrophic bacteria that can be found in several diverse habitats such as soil, root nodules and aquatic environments. The genus *Cupriavidus* belongs to the family *Burkholderiaceae* and the class *β-proteobacteria*. Remarkable heavy metal tolerance of environmental isolates has been confirmed [2, 3] and some species have important xenobiotic degradation potential such as *C. necator,* which is able to degrade chlorinated aromatic compounds [4–6]. An environmental strain, *C. basilensis* ŐR16 can degrade 98% of ochratoxin-A [7] (Table 1). One strain from the *C. taiwaniensis* species can initiate root nodule formation and nitrogen fixation [8]. A member of *C. respiraculi* was isolated from the respiratory tract of a cystic fibrosis patient [1].

**Table 1:** Xenobiotic biodegradation ability of the *Cupriavidus* genus strains

Species	Strain	Isolation matrix	Biodegraded chemicals	References
*C. necator*	NH9	Contaminated soil, Japan	Chlorinated aromatic chemicals; halo benzoate and nitrophenols	[9, 10]
	JMP134	Soil, unknown	2,4-D	[6]
*C. numazuensis*	TE26^T^	Natural soil, Japan, Numazu city, Shizuoka prefecture	Trichloroethylene, cis-dichloroethylene and toluene	[11]
*C. basilensis*	HMF14	Soil, Netherlands	Hydroxymetyl-furfural (HMF)	[12]
	JF1	BPA-degrading planted fixed-bed reactor	Bisphenol-A	[13]
	M91-3	Agricultural soil	Atrazine	[14]
	R25C6	PCP-contaminated soil, Ljungby, Sweden	Chlorobenzene, phenol	[15]
	B-8	Erosive bamboo slips, China	Kraft lignin biodegradation	[16]
	ÖR16	Natural soil, Hungary	Ochratoxin-A	[7]
	RK1	Freshwater pond, France	2,6-dichlorophenol	[17]
*C. gilardi*	CR3	Rancho La Brea Tar Pits 91, Los Angeles	Naphthenic acids	[18]
*C. pauculus*	KF709	Biphenyl-contaminated soil in Kitakyushu, Japan	Biphenyl	[19]
*C. nantongensis*	X1^T^	Sludge, chlorpyrifos manufacture plant Nantong, China	Chlorpyrifos	[20]
*C. pampae*	*CPDB6*^T^	Agricultural soil, Argentinean Humid Pampa region	2,4-D	[5]

To date, 11 *Cupriavidus* genome projects are known for the following strains, namely *C. necator* CCUG 52238^T^*, C. metallidurans* CCUG 13724^T^*, C. pinatubonensis* DSM19553^T^*, C. alkaliphilus BCCM* 26294^T^*, C. basileneis* DSM 11853^T^*, C. oxalaticus* JCM 11285^T^*, C. pauculus* JCM 11286^T^*, C. taiwanensis* CCUG 44338^T^*, C. campinensis* CCUG 44526^T^, *C. nantongensis* KCTC 42909^T^ and *C. plantarum* BCCM/LMG 26296^T^. The genome size of the genus varies from 6.5 to 8.5 Mbp [21]. Genomic sequences suggest that the species has significant catabolic potential, as several pathways responsible for aromatic ring cleavage have been identified, such as the catechol and protocatole ortho-ring cleavage, catechol meta-position ring cleavage, gentisate and benzene-CoA pathways [22] (Table 1).

**Fig. 1:**
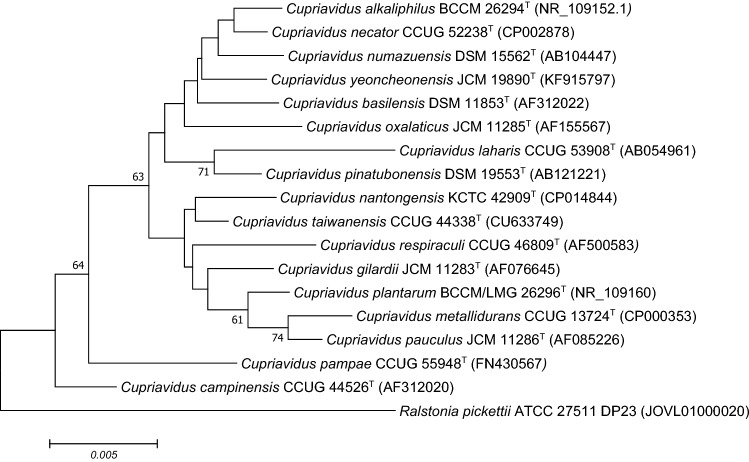
Neighbour-joining tree based on 16S rRNA gene sequences showing the phylogenetic relations of 17 type strains of *Cupriavidus* genus with AFB1, OTA, ZEA and T-2 detoxification ability. Bootstrap values are presented as percentages of 1000 replicates. Only values above 50% were shown. The tree analysis was conducted in MEGA7 software

The genus has 17 type strains, from which the biodegradation and detoxification potential of 16 has been investigated for five mycotoxins, namely aflatoxin B1 (AFB1), ochratoxin-A (OTA), zearalenone (ZON), T-2 toxin (T-2) and deoxynivalenol (DON). Figure 1 depicts the phylogenetic tree of the *Cupriavidus* genus type strains.

Contamination of food and feed by toxigenic moulds (fungi) is an increasing and unavoidable problem because the climatic extremities cause permanent stress for the crops, which becomes vulnerable to fungi. This leads to an increase in the number of mycotoxin contaminations amongst foodstuff globally [23, 24]. However, there are approximately 300 to 400 different identified mycotoxins. In the present study, five toxins will be investigated because they have a negative influence on human and animal health, such as genotoxicity, oestrogenity, nephrotoxicity and teratogenicity.

A limited number of methods have been developed to degrade mycotoxins and/or reduce their toxicity. Ozonation [25] can be applied as a chemical method. Sorting, extrusion and application of adsorbents are physical techniques [26] whilst biological methods include the biodegradation by microorganisms or their enzymes. The treatment of feed products by adsorbents such as bentonite and clay can efficiently bind toxins [27] although in the animal intestinal tract, these agents can also adsorb vitamins and nutrients [28].

The biodegradation of mycotoxins by different bacteria is a widely used method for decreasing their concentrations. The biodegradation is not synonymous with biodetoxification, and this phenomenon should be investigated in all cases of biodegradation. During the biodegradation, the by-product can be more toxic or harmful then the initial chemical. However, the investigation of the by-product toxicity is not straightforward. Biotests are useful for evaluating the detoxification efficiency and prior to making any product suitable for the market, detoxification from different biotests or assays is advised to determine the difference between the sensitiveness and behaviour of the test organism. However, detoxification is an appropriate way of treating contaminated feed and foodstuff.

## Materials and Methods

### *Cupriavidus* Strains and Mycotoxin Stock Solutions

Sixteen type strains of the genus *Cupriavidus* were purchased from the following strain collections, namely the Deutsche Sammlung von Mikroorganizmen und Zellculturen (DSMZ) (German Collection of Microorganism and Cell Cultures), the Japan Collection of Microorganisms (JCM), the Culture Collection University of Gothenburg (CCUG), Sweden and the Belgian Coordinated Collection of Microorganisms (BCCM). The 17th type strain *C. nantongensis* KCTC 42909^T^ from South Korea could not been ordered in time for the experiments. Mycotoxins were purchased from Sigma Aldrich Limited (Budapest, Hungary). From these mycotoxins, 1 mg/mL of stock solutions were prepared in acetone and used in degradation experiments.

### Chemicals and Reagents

With regards to the HPLC–MS/MS analysis, all standards and reagents were purchased from the Hungarian distributors of national companies. Mycotoxin standards with minimum 98% purity were purchased from Romer Labs (Hungary). Mobile phases and extraction solvents were used containing super gradient grade acetonitrile (MeCN, 99%) purchased from VWR (Hungary), gradient grade methanol (CH_3_OH, 99%) obtained from Fischer Scientific (Budapest), ultra-pure-grade water (18MΩcm) produced in-house by a Milli-Q water purification system (Merck, Darmstadt, Germany). Acetic acid (CH_3_COOH, 99.8%) obtained from Merck (Hungary), formic acid (HCOOH, 98%) from Scharlau (Hungary) and LC/MS grade ammonium-acetate (CH_3_COONH_4_, > 99%) from VWR (Hungary). Luria–Bertani (LB) media and acetone were ordered from BioLab Incorporated and Reanal Ltd Hungary.

### Biodegradation Experiments

*Cupriavidus* strains were stored at − 80 °C and were streaked on LB agar plates (10 g tryptone, 5 g yeast extract, 9 g NaCl and 18 g bacteriological agar dissolved in 1000 mL distilled water). The plates were incubated at 28 °C for three days for colony forming. A single colony was inoculated into Erlenmeyer flasks containing 50 mL liquid TGE-5 medium (5 g tryptone, 5 g glucose and 2.5 g yeast extract dissolved in 1000 mL distilled water). The flasks were incubated at 28 °C (Sartorius AG, Germany). After three days, the optical density of the cultures was measured by an UV–Vis spectrophotometer (Genesys 10 UV–Vis, Thermo Fisher Scientific Incorporated, US) and adjusted to OD600 = 1.0. From this culture, 5 mL was inoculated into 45 mL freshly sterilized LB medium, which was inserted with the stock solution of the mycotoxins, 1 µg/mL initial concentration in each mycotoxin. The monotoxins experiment was conducted in triplicates. 50 mL sterile LB medium containing the mycotoxin was applied as a microbe-free control. Flasks were incubated at 28 °C for five days, thereafter, 1 mL samples were taken and centrifuged at 20,800×*g* for 15 min (Eppendorf 5810R Centrifuge, Eppendorf, Germany). Supernatants and pellets were separated and stored at − 80 °C for further HPLC MS/MS analysis.

### Genotoxicity Test

The genotoxic effect in supernatant samples was observed by the colorimetric SOS-chromotest (Environmental Bio-Detection Products Incorporated, Canada). In the genetically modified test organism, *E. coli* PQ37, operon fusion of *sfiA* and *lacZ* genes was conducted. As a result, when the SOS-repair mechanism commenced, *β-galactosidase* was produced simultaneously, which was proportional to the strength of the genotoxicity [29]. The test was conducted according to the description of Risa et al*.* [30]. The genotoxic effect was expressed in induction factor (IF), which was calculated according to Eq. (1) [31]:

1 $${\text{Induction factor }}\left( {{\text{IF}}} \right) = ({\text{C4}}0{5} * {\text{S62}}0)/({\text{S4}}0{5} * {\text{C62}}0)$$

where* C* is the mean of the absorbance value of the control and S is the mean of the absorbance value of the sample measured at 405 and 620 nm wavelength.

Samples were considered as not genotoxic, when IF was significantly (*p* < 0.05) less than 1.5 [32].

### Oestrogenicity Test

With regards to the detection of the oestrogenic effect of ZON and its metabolites, the bioluminescence-based BLYES test was used [33]. The *Saccharomyces cerevisiae* BLYES strain had been genetically modified, inserting gene encoding human estrogenic receptor, *lux* genes and estrogenic response element into its genome. This modification made the BLYES strain capable of emitting light after an oestrogenic compound binds to the oestrogenic receptor. The test was conducted in accordance with Risa et al. [31]. The oestrogenic effect was expressed in bioluminescence intensification (%), which was calculated according to Eq. (2) [34]:

2 $${\text{Bioluminescence intensication }}\left( \% \right) = \left( { - 1} \right)*(({\text{C}} - {\text{S}})/{\text{ C}})*100$$

where* C* is the mean of the bioluminescence values of the negative control and S is the mean of the bioluminescence values of the sample.

### Zebrafish Microinjection Tests for OTA and T-2

#### Preparation of the Bacterial Inocula

Bacterial inocula (5 mL) was prepared as stated and added to 45 mL 20% LB medium containing OTA and T-2 separately (monotoxic) (7 mg/L final concentration). Similar inocula were prepared in parallel without mycotoxins to test the effects of bacterial metabolites. Uninoculated LB medium (20%) was combined with OTA and T2 (7 mg/L) was used as a negative control. Both of the cultures and controls were incubated at 28 °C, 170 rpm for 120 h in triplicates. After the incubation, cultures were centrifuged at 14,000×*g*, 4 °C for 15 min. Supernatants were filtered with 0.2 µm syringe filters, and samples were stored at − 20 °C until microinjection.

### Microinjection

#### Animal Protection

The animal protocol was approved under the Hungarian law regarding animal welfare (XIV-I-001/2303-4/2012) and all studies were completed before the treated individuals would have reached the free feeding stage.

#### Zebrafish Maintenance and Egg Collection

Laboratory-bred wild type AB strain zebrafish were held in breeding groups of 30 females and 30 males at the Department of Aquaculture, Szent István University, Hungary, in a Tecniplast ZebTEC recirculation system (Tecniplast S.p.A., Italy) at 25.5 °C ± 0.5 °C, pH 7.0 ± 0.2, conductivity 550 ± 50 µS (system water) and a light: dark period of 14 h:10 h. The fish were fed twice a day with dry granulate food (Zebrafeed 400–600 µm, Sparos Lda., Portugal) supplemented with freshly hatched live Artemia salina once a day. The fish were placed in breeding tanks (Tecniplast S.p.a.) late in the afternoon the day before the experiment and allowed to spawn by removing the dividing walls the following morning. Spawning of individual pairs was delayed to allow for a continuous supply of 1-cell embryos.

#### Microinjection

Microinjection was conducted as described by Csenki et al*.* [35]. The changes were in the volume of injection, namely the sphere diameter of 50 µm corresponded to an injection volume of 0.074 nL, 75 µm to 0.22 nL, and 200 µm to 4.17 nL.

#### Experimental Design

Bacteria metabolites were injected with 4.17 nL volume in three replicates (20 eggs per replicate). 7 mg/L T-2 toxin and bacteria T-2 toxin degradation products were injected with 0.074 nL and 4.17 nL volumes in three replicates (20 eggs per replicate). 7 mg/L OTA toxin and bacteria OTA toxin degradation products were injected with 0.22 nL and 4.17 nL volumes in three replicates (20 eggs per replicate).

### Examination of Injected Embryos

Embryo mortality was determined at 120 hpf based on egg coagulation, the lack of somite formation and the lack of heart function.

### Analytical Measurements

High-performance liquid chromatography with tandem mass spectrometry (HPLC–MS/MS) was conducted for the measurement of AFB1, ZON, OTA, T-2 and DON concentrations. From the three experiments’ culture, lombics 1–1 mL inocula was taken as a sample, centrifuged for 20 min at 4000 rpm, and the supernatant and pellet separated. In terms of the supernatants, 500 μL was transferred into 1.5 mL vials, then evaporated, reconstituted with A/B eluent (50:50%) and injected directly into the HPLC–MS/MS. In terms of the pellet 1 mL extraction, solvent acetonitrile/water/formic acid was added and vortexed for 1 min. Subsequently, 500 μL of suspension was transferred to 1.5 mL vials then handled as supernatants. 500 μL samples were evaporated until dry under a gentle N2 stream. Thereafter, it was reconstituted in 50–50 V/V% A-B eluent (A: water, 5 mM ammonium-acetate, 0.1% acetic acid; B: methanol, 5 mM ammonium-acetate, 0.1% acetic acid) and filtered through a 0.22 µm PTFE syringe filter. With regards to the chromatographic separation, an Agilent 1100 HPLC (Agilent Technologies, US) equipped with Agilent Zorbax C18 column (3.5 µm, XDB-C18, 2.1 × 50 mm) was used. 10 μL prepared samples were injected into the mobile phase containing A–B eluent. 400 µLmin-1 flow rate and 40 °C column temperature was set. The 3200 QTRAP LC/MS/MS system (Applied Biosystems, US) was equipped with a Turvo V electrospray ionization (ESI) interface in positive (DON, T2, AFB1, OTA) and negative (ZON) ion mode was used. Prior to the HPLC–MS/MS measurements, a method was developed and validated. The mycotoxins were separated on a reverse phase C18 chromatographic column. Two methods were used, the first method included DON, T2, AFB1 in positive ion mode and ZON in negative ion mode; whist the method included OTA in positive ion mode. The AFB1 and OTA were co-eluting, thus, the researchers chose to measure OTA separately with the same analytical condition as in case of the first method. The chromatograms of the standards are depicted in Fig. S1 (A) and (B). The validation was performed on LB medium. Recoveries for all compounds were between 70 and 114% with an RSD < 20%. The matrix effect (ion suppression/enhancement) which is the result of the competition between non-volatile matrix components and analyte ions in ESI ion source has also been studied. These effects were between − 37 and + 10%, thus, a matrix matched linear calibration curve was used for quantification. LOD values were between 0.2 and 5 µg/kg and LOQ between 1 and 15 µg/kg for all studied mycotoxins. The validation parameters of HPLC–MS/MS method can be observed in Table. S2. The mycotoxin degrading potential of investigated *Cupriavidus* strains were confirmed by low resolution target HPLC–MS/MS methods. With this instrument, only native mycotoxins with available standards can be qualitatively and quantitatively measured. The metabolomic profile of mycotoxin by-products can be identified with high resolution MS, in further study.

### Statistical Analysis

The statistical analysis was performed with Microsoft Excel 2016 (Microsoft Office, Microsoft Incorporated, US), Past 3 [36] and GraphPad Prism 6.01 (GraphPad Software, San Diego, US). Biological effects were expressed in induction factor (Eq. 1), bioluminescence intensification percent (Eq. 2) and mortality ratio. The residual toxin concentrations determined by HPLCFLD were expressed in ng/mL. Data were checked for normality with the Shapiro–Wilk normality test and non-compliance with the requirements of parametric methods was established. Significant differences (*P* < 0.05) were verified by one-sample t-tests and Kruskal–Wallis analysis with the Dunn’s multiple comparisons test. All values are means of the triplicates. Correlations between biotests and analytical measurements were calculated by Spearman’s rank correlation coefficient.

## Results

### Biodegradation of AFB1

Residual AFB1 concentrations were measured by HPLC–MS/MS (Table 2). Four type stains had excellent biodegradation potential. Remarkable (91%) biodegradation of AFB1 was observed in the case of *C. laharis* CCUG 53908^T^. High AFB1 degradation rates (72–82%) were demonstrated by *C. oxalaticus* JCM 11285^T^, *C. metallidurans* CCUG 13724^T^ and *C. numazuensis* DSM 15562^T^ whilst moderate AFB1 reduction (approximately 60%) was observed in five cases, namely *C. taiwanensis* CCUG 44338^T^, *C. campinensis* CCUG 44526^T^, *C. pampae* CCUG 55948^T^, *C. plantarum* BCCM/LMG 26296^T^ and *C. alkaliphilus* BCCM/LMG 26294^T^. Two strains could detoxify AFB1 for five days according to the SOS-chromotest, *C. laharis* CCUG 53908^T^ and *C. oxalaticus* JCM 11285^T^ (*p* < 0.05) (Fig. S3).

**Table 2:** AFB1, ZON, OTA and T-2 biodegradation potential of *Cupriavidus* type strains after a 5 day-experiment determined by HPLC–MS/MS

Species	AFB1 biodegradation efficiency (%)	Genotoxicity (IF) Day 5	ZON biodegradation efficiency (%)	Oestrogenicity (Biol. int. %)	OTA biodegradation efficiency (%)	T2 biodegradation efficiency (%)
*Cupriavidus alkaliphilus* BCCM 26294^ T^	58	2.83 ± 0.14	33	1053 ± 110	**95**	52
*Cupriavidus basilensis* DSM 11853^ T^	19	2.71 ± 0.29	**96**	*47* ± *19*	**94**	68
*Cupriavidus campinensis* CCUG 44526^T^	61	2.60 ± 0.09	55	894 ± 187	28	55
*Cupriavidus gilardii* JCM 11283^T^	32	3.45 ± 0.34	35	1140 ± 9	19	**95**
*Cupriavidus laharis* CCUG 53908^T^	**91**	*1.31* ± *0.03*	61	811 ± 6	20	27
*Cupriavidus metallidurans* CCUG 13724^T^	**77**	2.27 ± 0.15	51	1084 ± 59	27	**73**
*Cupriavidus necator* CCUG 52238^T^	31	3.44 ± 0.26	47	1092 ± 14	**92**	47
*Cupriavidus numazuensis* DSM 15562^T^	**72**	1.93 ± 0.11	**85**	530 ± 16	**85**	**70**
*Cupriavidus oxalaticus* JCM 11285^T^	**82**	*0.97* ± *0.14*	**82**	541 ± 21	19	50
*Cupriavidus pampae* CCUG 55948^T^	60	2.82 ± 0.10	50	772 ± 101	30	47
*Cupriavidus pauculus* JCM 11286^T^	41	3.04 ± 0.51	42	1118 ± 90	20	42
*Cupriavidus pinatubonensis* DSM 19553^T^	17	2.90 ± 0.28	**91**	312 ± 50	**88**	68
*Cupriavidus plantarum* BCCM/LMG 26296^T^	59	2.81 ± 0.12	67	911 ± 177	14	60
*Cupriavidus respiraculi* CCUG 46809^T^	51	3.22 ± 0.12	64	310 ± 34	**82**	47
*Cupriavidus taiwanensis* CCUG 44338^T^	63	2.93 ± 0.20	42	636 ± 215	**97**	56
*Cupriavidus yeoncheonensis* JCM 19890^T^	45	3.34 ± 0.32	41	1232 ± 36	12	40

Bold values indicates strains having more than 70% biodegradation ability

Italic values indicates strains causing biodetoxification

Residual genotoxicity was detected in supernatant by SOS-Chromo test, oestrogenicity was detected by BLYES test

In the pellet fraction of *C. laharis* CCUG 53908^T^ 120 ng/mL (12% of the initial toxin concentration) and *C. oxalaticus* JCM 11285^T^ 247 ng/mL (24% of the initial toxin concentration), AFB1 was measured. Adsorption was observed in other cases also and the amount varied between 50 and 254 ng/mL AFB1 in the pellet as the biodegradation potential was corrected by the residual toxin concentration on the pellet.

### Biodegradation and Detoxification of ZON

According to the analytical results (Table 2), the highest ZON biodegradation rates (82–96%) were detected in the case of *C. basilensis* RK1 DSM 11853^T^, *C. pinatubonensis* DSM 19553^T^, *C. numazuensis* DSM 15562^T^ and *C. oxalaticus* JCM 11285^T^.

In terms of ZON degradation, the highest toxin concentration on the cell pellet was 155 ng/mL (15% of the initial toxin concentration), which was observed in the *C. oxalaticus* JCM 11285^T^ strain, which demonstrated a high degradation ability (82%). In terms of *C. basilensis* DSM 11853^T^, the pellet ZON concentration was the lowest at 10 ng/mL. The ZON in the pellet varied between 10 and 155 ng/mL as the biodegradation potential was corrected by the residual toxin concentration on the pellet.

In order to analyse cytotoxicity and the oestrogenic effect of ZON BLYES, tests were performed, respectively. According to the BLYES test (Fig. S4), a considerable reduction of the oestrogenic effect of ZON was observed in the strain *C. basilensis* DSM 11853^T^ (98%) compared to the control, which demonstrated a positive correlation with the biodegradation rate in the HPLC analysis. Biodetoxification occurred in the following cases: *C. respiraculi* CCUG 46809^T^ reduced the oestrogenity (73%) by the 5th day, although the degradation was only 64% as measured by HPLC, which is an extremely effective degradation and detoxification ratio. The *C. numazuensis* DSM 15562^T^ and *C. oxalaticus* JCM 11285^T^ could decrease the oestrogenic effect by 50%, with an 85% biodegradation ratio. *C. pinatubonensis* DSM 19553^T^ reduced the oestrogenic effect of ZON to approximately 30% with a 91% biodegradation rate by day five. Four strains, *C. necator* CCUG 52238^T^, *C. gilardii* JCM 11283^T^, *C. pauculus* JCM 11286^T^ and *C. yeoncheonensis* JCM 19890^T^ had a higher bioluminescence rate (up to 118%) compared to the control which leads to the transformation of ZON in additional oestrogenic metabolites.

### The Biodegradation of OTA

Residual OTA concentrations in the supernatant and pellet samples were measured by HPLC–MS/MS. The OTA degradation potential of *Cupriavidus* type strains differ significantly. Out of the 16 type strains, six strains were the most effective. *C. taiwanensis* CCUG 44338^T^ demonstrated the highest OTA-reduction rate (97%), *C. alkaliphilus* BCCM 26294^T^ showed 95%, *C. basilensis* RK1 DSM 11853^T^ showed 94%, *C. necator* CCUG 52238^T^ showed 92%, *C. pinatubonensis* DSM 19553^T^ showed 88%, *C. numazuensis* DSM 15562^T^ showed 85% and *C. respiraculi* CCUG 46809^T^ showed 82%. Measuring OTA-binding to the cells confirmed that adsorption was negligible. The highest OTA concentration on the cell pellet (7 ng/mL, 0.7% of the initial toxin concentration) was observed in case *C. numazuensis* DSM 15562^T^ strain, with a degradation ability of 85%.

### Biodegradation of Trichothecene Mycotoxins

#### Biodegradation of T-2

Out of the 16 type strains, six were able to degrade T-2. The highest biodegradation rate was 95%, which was observed in the case of the *C. gilardi* JCM 11283^T^ strain. Five strains demonstrated a moderate (68–88%) T-2 biodegradation rate, namely *C. metallidurans* CCUG 13724^T^, *C. numazuensis* DSM 15562^T^, *C. pinatubonensis* DSM 1955^T^, *C. basilensis* DSM 11853^T^ and *C. plantarum* LMG 26296^T^. The T-2 concentration in the pellets was between 22 and 50 ng/mL, this is 2% and 5% of the initial toxin concentration, respectively.

#### Biodegradation of DON

According to the analytical results, none of the 16 type strains could degrade DON.

#### Ability to Degrade More than one Mycotoxin

From the 16 type strains, six were able to degrade 60% of the two different mycotoxins. The *C. respiraculi* CCUG 46809^T^ strain could degrade 82% of OTA and 64% of ZON. *C. laharis* CCUG 53908^T^ strain could degrade 91% of AFB1 and detoxificate the harmful effects of the metabolites as well as degrade 61% of ZON. The *C. metalliduriens* CCUG 13724^T^ strains could degrade 77% of AFB1 and 72% of T-2. The *C. plantarum* BCCM/LMG 26296^T^ strain could degrade 67% of ZON and 60% of T-2. The *C. taiwanensis* CCUG 44338^T^ strain could degrade 97% of OTA and 63% of AFB. The *C. oxalaticus* JCM 11285^T^ strain could degrade 82% of AFB1 and 82% of ZON, furthermore, it was able to eliminate the genotoxic effect of AFB1 and its by-products.

*The C. pinatubonensis* DSM 19553^T^ strain was able to degrade 90% of ZON, 88% of OTA and 68% of T-2. The *C. basilensis* DSM 11853^T^ strain could degrade 96% of ZON, 94% of OTA and 68% of T-2, moreover, in the case of ZON. it could eliminate the oestrogenic effect of the metabolites. The *C. numazuensis* DSM 15562^T^ strain was the most effective in the degradation of mycotoxins, as it reduced 70% of T-2, 72% of AFB1, 85% of ZON and 85% of OTA. In order to investigate the detoxification efficiency of three multi-degrading strains, the zebrafish embryo microinjection test was conducted to assess the by-products of OTA and T-2.

### Zebrafish Embryo Microinjection Test for Evaluating the by-Products of OTA and T-2

In order to evaluate the biodetoxification in terms of T-2 and OTA, the multi mycotoxin degraders *C. numazuensis* DSM 15562^T^, *pinatubonensis* DSM 19553^T^ and *basilensis* DSM 11853^T^ were measured with a newly developed and standardised zebrafish embryo microinjection test method (Csenki et al*.* 2019). The strains were selected for this method because the *C. numazuensis* DSM 15562^T^ can degrade four mycotoxins (AFB1, ZON, T-2 and OTA) whilst the *C. pinatubonensis* DSM 19553^T^ and *basilensis* are able to degrade three mycotoxins (ZON, T-2 and OTA).

According to the microinjection results of the normal metabolites, the strains are toxic for the embryos, with a 60% mortality ratio indicating a high toxicity level. The source of the strain’s toxicity is currently unknown. According to the existing literature, there are no data on the pathogenicity of the *Cupriavidus* genus amongst fish. The injected supernatants were cell free, without any bacteria, only the centrifuged-filtered inocula containing the by-products and metabolites of the strains. The pellets of the inocula were also investigated by the HPLC, where the residual T-2 concentration was only 2–5% of the initial toxin concentration.

**Fig. 2:**
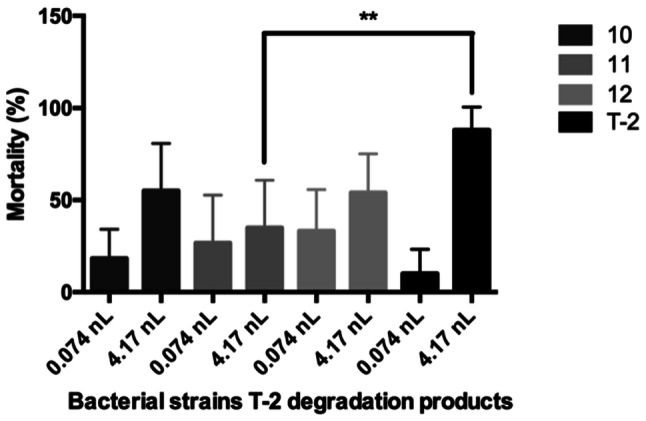
Bacterial strains T-2 degradation products were injected with 0.074 nL and 4.17 nL volumes. All strains degradation products were toxic on zebrafish embryos. Statistically significant differences were detected between 11 strain and T-2 (*p* < 0.01) at 4.17 nL injected volume. Number 10 was *C. numazuenzis* DSM 15562^T^, 11 was *C. pinatubonensis* DSM 19553^T^, 12 was *C. basilensis* DSM 11853^T^ type strain

**Fig. 3:**
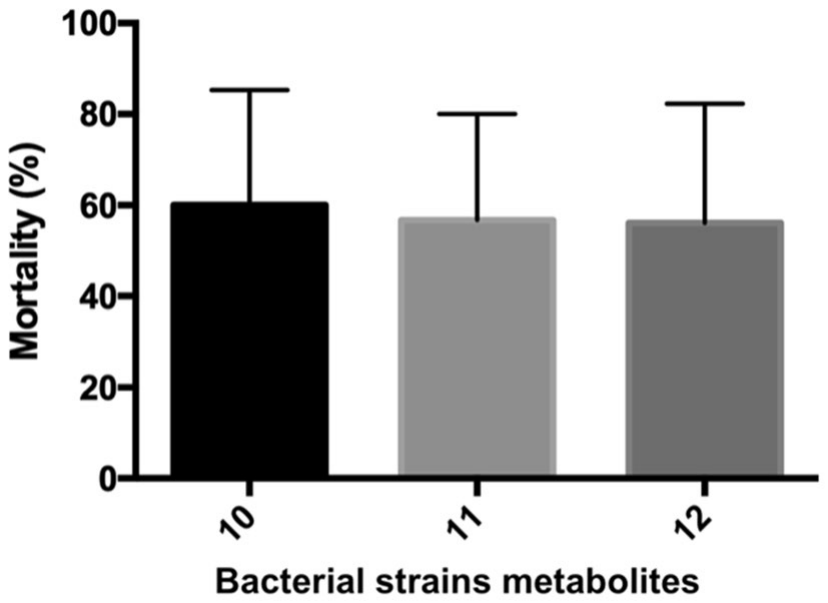
Bacterial strains metabolites were injected with 4.17 nL volume. All strains were toxic on zebrafish embryos. Statistically significant differences were not detected between strains. Number 10 was *C. numazuenzis* DSM 15562^T^, 11 was *C. pinatubonensis* DSM 19553^T^, 12 was *C. basilensis* DSM 11853^T^ type strain

With regards to the T-2 microinjection test, *C. pinatubonensis* DSM 19553^T^ demonstrated a significant reduction at 4.17 nL injected volume (*p* < 0.01) in the mortality compared to the 7 mg/L T-2 control. Although the bacteria strain reduced the lethality rate, the degraded metabolites were toxic to zebrafish (mortality rate: 40%) (Fig. 2). The effect of the T-2 metabolites ratio was less than the metabolites effect revealed in Fig. 3.

**Fig. 4:**
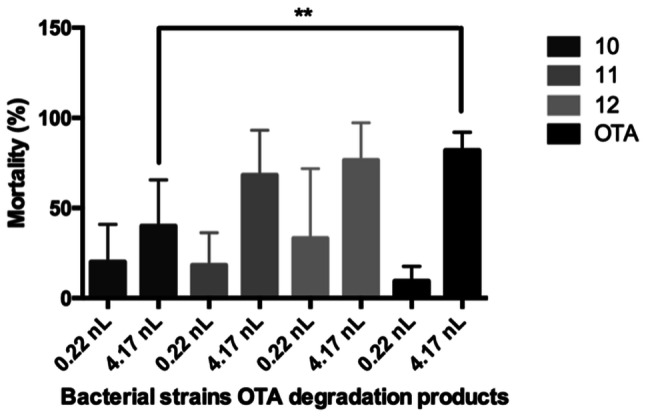
Bacterial strains OTA degradation products were injected with 0.22 nL and 4.17 nL volumes. All strains degradation products were toxic on zebra fish embryos. Statistically significant differences were detected between 10 strain and OTA (*p* < 0.01) at 4.17 nL injected volume. Number 10 was *C. numazuenzis* DSM 15562^T^, 11 was *C. pinatubonensis* DSM 19553^T^, 12 was *C. basilensis* DSM 11853^T^ type strain

In terms of the OTA microinjection test, *C. numazuensis* DSM 15562^T^ demonstrated a significant decrease at 4.17 nL injected volume (*p* < 0.01) in the mortality rate compared to the 7 mg/L OTA control. Although the bacteria strain reduced the lethality rate, the degraded metabolites were toxic to zebrafish (mortality rate: 45%) (Fig. 4). The effect of OTA metabolites ratio was less than the metabolites effect depicted in Fig. 3.

## Discussion

In the present study, the aim was to measure the mycotoxin biodegradation potential of 16 type strains of *Cupriavidus* genus and evaluate the potential harmful effects of the metabolic intermediates. The research also aimed to select the best degraders amongst these strains.

The mycotoxin degradation ability of different bacteria (*Rhodococcus* sp, *Streptomyces* sp) was previously investigated by this department [30, 37, 38]. Members of the *Cupriavidus* genus have profound abilities in terms of the biodegradation of different chemicals and xenobiotics, particularly one mycotoxin OTA [7]. The biodegradation experiments were performed within five days because of the comparison of the aforementioned existing studies. Although the biodegradation result was confirmed after two or three days and the researchers also realised the 95% AFB1 biodegradation rate for 24 h, the present study is the first evaluation of the *Cupriavidus* genus biodegradation ability in terms of mycotoxins.

The comparison of the biodegradation potential of different bacteria is not the most appropriate foundation because biodegradation does not necessary signify biodetoxification, which refers to the elimination of the harmful effects of the biodegraded chemical. The comparison of the biodetoxification ability is an appropriate method. However, evaluation of the biodetoxification is challenging because an appropriate biotest or organism is required. Mycotoxins have different negative effects, which are not easy to measure or estimate realistically due to cost effectiveness, time and resources. Unfortunately, only limited publications have investigated biodetoxification in terms of biodegradation.

In terms of AFB1, *Rhodococcus* sp are highly effective biodetoxifiers from 42 type strains of which 15 terminated the genotoxicity in 72 h. With regards to ZON, only one *Rhodococcus* type strain could terminate the eostrogenic effect [30]. 124 Streptomyces strains were tested for AFB1 and ZON biodegradation, and only one strain was able to biodetoxify AFB1 and only two strains could terminate the eostrogenic effect of ZON [38].

From the genus *Cupriavidus,* almost all the type strains are able to biodegrade AFB1 in five days and a high biodegradation ratio (over 70%) was achieved by only four strains, namely *C. laharis* CCUG 53908^T^ (91%), *C. oxalaticus* JCM 11285^T^ (82%), *C. metallidurans CCUH 13724*^*T*^ (77%) and *C. numazuensis* DSM 15562^T^ (72%). According to the SOS-chromotest results, two strains were able to terminate the genotoxicity. These were *C. laharis* CCUG 53908^T^ (IF = 1.31) and *C. oxalaticus* JCM 11285^T^ (IF = 0.97). The biodegradation and biodetoxification was observed, and absorption on the pellet was not assuming an integral role as in the case of the elimination of AFB1. In Fig. S3, high degradation ratios can be observed. In the absence of the eliminations of the genotoxic effect in the case of 14 strains, all degradation results were corrected with the residual AFB1 concentration measured on the pellets. Only the aforementioned *C. laharis* CCUG 53908^T^ and *C. oxalatixus* JCM 11285^T^ were able to terminate the genotoxicity.

In the case of ZON, all *Cupriavidus* strains had biodegradation ability, but only four attained a 70% rate, namely *C. basilensis* DSM 11853^T^ (95%), *C. pinatubonensis* DSM 19553^T^ (91%), *C. numazuensis* DSM 15562^T^ (85%) and *C. oxalaticus* JCM 11285^T^ (82%). According to the BLYES results, one strain was able to reduce the endocrine disrupting effect of the metabolites of ZON, namely the C*. basilensis* RK1 DSM 11853^T^ strain (Fig. S4).

Comparing the results of the present study with the *Rhodococcus* genus ability, the genus *Cupriavidus* has less appropriate members for detoxifying the mycotoxins, but still a valuable resource for further research and for future application against mycotoxins.

From the 16 type strains, in the case of OTA, there were two groups, namely strains with a weak biodegradation ability (30%) and strains with a remarkable biodegradation potential (over 80%). The most effective strains were *C. taiwanensis CCUG 44338*^*T*^ (97%), *C. alkaliphilus BCCM 26294*^*T*^ (95%), *C. basilensis* (94%), *C. necator* (92%), *C. pinatubonensis* (88%), *C. numazuensis* (85%) and *C. respiculi* (82%). The evaluation of the biodetoxification ability of theses strains are limited, because there are only difficult and time-consuming biotests or methods for testing the negative effects of the OTA by-products. In the case of the *Cupriavidus basilensis* ÖR16 wild strain, which has a remarkable 98% OTA biodegradation and biodetoxification ability, the first biotest was a mice feeding experiment performed by Ferenczi et al*.* in 2014. This method took approximately two months with a 21-day long feeding experiment, and one month for evaluating the results on the level of gene expression, weight and biopsy. ÖR16 strain was biodetoxifing the OTA effectively in five days. No harmful effects were observed. The ÖR16 strain was also tested by a novel Danio rerio embryo microinjection method developed by Csenki et al*.* [35], the test required approximately seven days to obtain the results. In this test, the strain by-products had the same effect as the results of this research and it caused 50% mortality amongst the embryos. However, the OTA biodegraded supernatant was less harmful than the OTA control and the normal by-product control.

The strain *C. basilensis* DSM 11853^T^ was tested for biodetoxification of OTA using a Danio rerio microinjection test. According to the results, it is not able to decrease the harmful effect of the OTA by-products, although it demonstrated a 94% toxin degradation. *C. numazuensis* DSM 15562^T^ was also evaluated by the microinjection test, where the strain OTA by-product had a significantly reduced harmful effect in comparison to the OTA containing control.

In terms of the T-2 toxin, only six strains could biodegrade T-2 with a ratio exceeding 60%, namely *C. gilardii* JCM 11283^T^ (95%), *C. metallidurans* CCUG 13724^T^ (73%), *C. numazuensis* DSM 15562^T^ (70%), *C. pinatubonensis* DSM 19553^T^ (68%), *C. basilensis RK1* DSM 11853^T^ (68%) and *C. plantarum* BCM/LMG 26296^T^ (60%). The evaluation of the T-2 detoxification has an identical problem with the OTA. In the case of the three strains, the researchers used the microinjection test for evaluation of the detoxification. In terms of the *C. pinatubonensis* DSM 19553^T^, the T-2 by-products were significantly less harmful than the T-2 control, according to the results. Biodegradation of DON was also investigated but none of the 16 type strains were able to degrade it.

From the 16 type strains, five strains were able to degrade two or more mycotoxins effectively (over 60%). The *C. metallidurans* CCUG 13724^T^ could biodegrade AFB1 and T-2. The *C. oxalaticus* JCM 11285^T^ could degrade AFB1 and ZON. The *C. pinatubonensis* DSM 19553^T^ and *C. basilensis* RK1 DSM 11853^T^ could degrade ZON, OTA and T-2. The *C. numazuensis* DSM 15562^T^ was able to biodegrade four mycotoxins, namely AFB1, ZON, OTA and T-2. This phenomenon is unique according to the existing literature. To date, *Rhodococcus* strains are known to degrade and detoxify more than two mycotoxins, for example, *R. erythropolis* NI1 strain can biodegrade AFB1, ZON and T-2 and detoxify the harmful effects of AFB1 and ZON [30]. A microbe consortia TMDC was investigated recently, which was able to simultaneously degrade AFB1 and ZEA in excess of 90% after 72 h but the detoxification was not evaluated. The consortia was comprised of the following generea *Geobacillus*, *Tepidimicrobium*, *Clostridium*, *Aeribacillus*, *Cellulosibacter*, *Desulfotomaculum* and *Tepidanaerobacter* [39].

The *C. pinatubonensis* DSM 19553^T^, *C. numazuensis* DSM 15562^T^ and C*. basilensis RK1* DSM 11853^T^ species were selected for a teratogenicity test using a Danio rerio embryo microinjection. According to the results, metabolites of these three strains were also toxic to the embryos and the mortality was 60% in all cases. This result resembles that of the *C. basilensis* ŐR16 wild strain [7] because the mortality was 50% in that case also. It appears that the members of the *Cupriavidus* genus have some toxic-by-product for fish embryos because the injected inocula was cell free. There is no information stating that *Cupriavidus* strains are pathogenic for fish.

In terms of the *C. numazuensis* DSM 15562^T^ strain, the harmful effect of the toxin by-products was significantly less than the OTA control. A similar result was observed with T-2 detoxification by the *C. pinatubonensis* DSM 19553^T^ strain. In both cases, the mortality ratio induced by the toxin breakdown products was less than with the bacterial metabolites.

Ultimately, according to the results, the *Cupriavidus* genus mycotoxin biodegradation ability could be a promising advantage in the future. At present, 11 type strains have genome project data. If all the members of the genus had full genome project data, it could be combined with the results of the present study and the responsible genes for mycotoxins biodegradation can be identified. This will help develop a cell free enzyme-based additive for treating the contaminated feed or crop.

The validation of the detoxification in the case of T-2 and OTA degrading members, and the investigation of the simultaneous mycotoxin degradation and detoxification should be implemented.

## Electronic supplementary material

Below is the link to the electronic supplementary material.

Supplementary file1 (DOCX 97 kb)
