# Supplementary material for: Mycotoxin Biodegradation Ability of the Cupriavidus Genus
Source: Curr Microbiol. 2020 Jun 5;77(9):2430–40. doi: 10.1007/s00284-020-02063-7 (PMC7415022; doi:10.1007/s00284-020-02063-7)
Supplement: Supplementary file 1 — Supplementary file1 (DOCX 97 kb) [file 284_2020_2063_MOESM1_ESM.docx]

**S1: (A).** Total ion chromatogram of DON (691 ng/ml), T2 (69.1 ng/ml), AFB1 (4.6 ng/ml) and ZON (103.7 ng/ml) in standard solution.

**
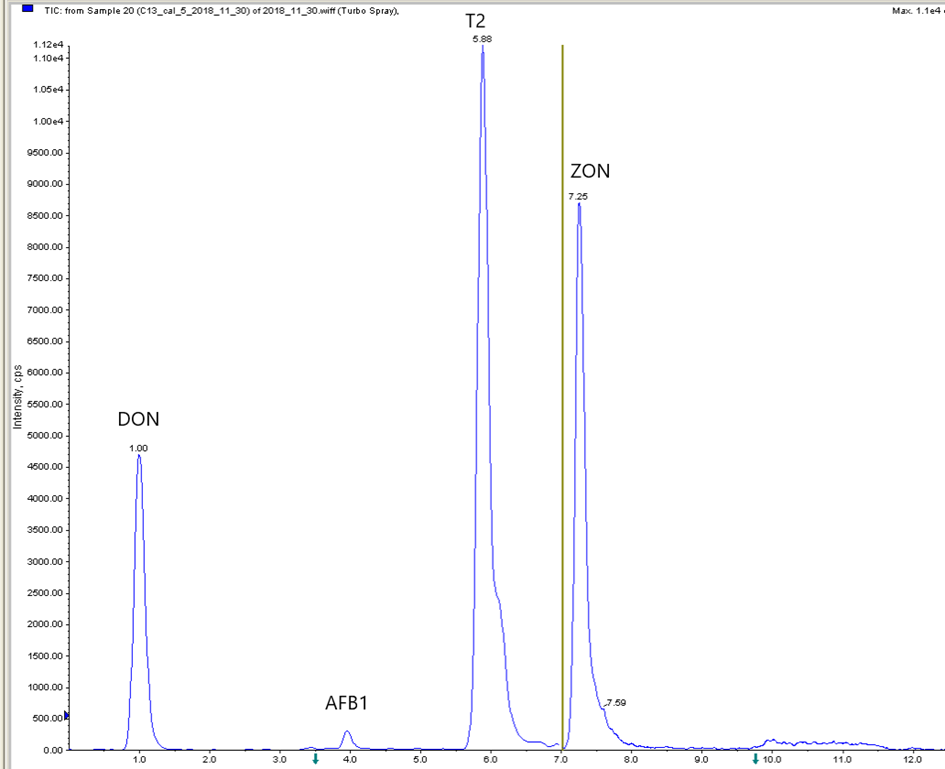
**

**S1 (B).** Total ion chromatogram of OTA (29.8 ng/ml) in standard solution.


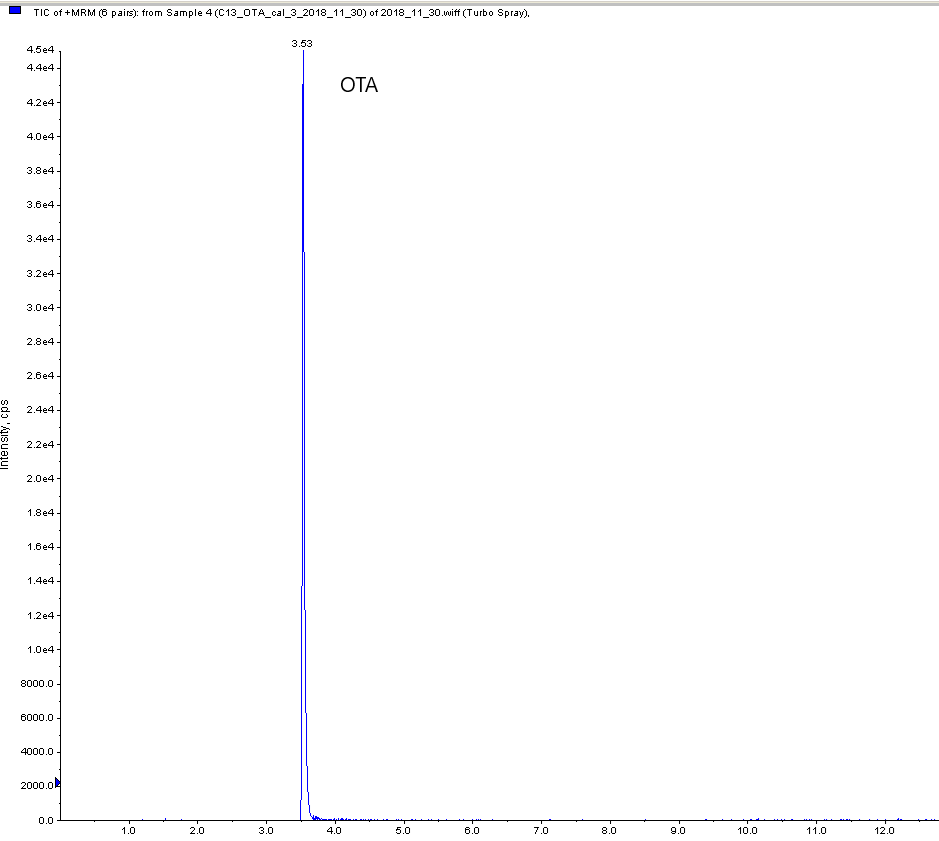


**Table S2 :** Validation parameters of HPLC-MS/MS method.

| **Mycotoxin** | **Correlation coefficient (R2)** | **Recovery % (RSD)** | **Matrix effect (%)** | **LOD (μg/kg)** | **LOQ (μg/kg)** |
| --- | --- | --- | --- | --- | --- |
| DON | 0.9977 | 70 (6.9) | -29 | 6 | 15 |
| T2 | 0.9936 | 78 (13) | 10 | 3 | 11 |
| AFB1 | 0.9181 | 114 (19) | -37 | 0.5 | 2 |
| OTA | 0.9927 | 80 (14) | -14 | 2 | 6 |
| ZEA | 0.9905 | 79 (4.2) | -17 | 0.2 | 1 |

**S3 :** Genotoxicity in supernatant samples of *Cupriavidus* type strains derived from AFB1 biodegradation experiment measured by SOS-Chromo test. Genotoxic effect was expressed in Induction Factor (IF). Values significantly less (p<0.02) than IF 1.5 indicates that the bacteria can detoxify AFB1.

**S4:** Oestrogenicity in supernatant samples of *Cupriavidus* type strains derived from ZON biodegradation experiment measured by BLYES test. Oestrogenic effect was expressed in bioluminescence intensification (%). Two ** means significant difference (p<0.02) compared to the control
